# Osborn's Tongue Holder and Duct Compressor

**Published:** 1873-04

**Authors:** 


*Osborn's Tongue Holder and Duct Compressor.*—An experience of nearly ten months with this appliance justifies us in saying that it has no equal; and we advise all who have not yet obtained it to do so, feeling satisfied that they will not regret the outlay. Like the Morrison engine, it has become indispensable in our practice, and for controlling the secretions of the sublingual and submaxillary glands, confining the tongue, and reflecting light upon the tooth to be operated on, it is certainly the most complete appliance in use.

